# Visuospatial characteristics of an elderly Chinese population: results from the WAIS-R block design test

**DOI:** 10.3389/fnagi.2015.00017

**Published:** 2015-02-25

**Authors:** Shufei Yin, Xinyi Zhu, Xin Huang, Juan Li

**Affiliations:** ^1^Center on Ageing Psychology, Key Laboratory of Mental Health, Institute of Psychology, Chinese Academy of SciencesBeijing, China; ^2^University of Chinese Academy of SciencesBeijing, China

**Keywords:** WAIS-R block design test, visuospatial characteristics, mild cognitive impairment, dementia, normative data

## Abstract

Visuospatial deficits have long been recognized as a potential predictor of dementia, with visuospatial ability decline having been found to accelerate in later stages of dementia. We, therefore, believe that the visuospatial performance of patients with mild cognitive impairment (MCI) and dementia (Dem) might change with varying visuospatial task difficulties. This study administered the Wechsler Adult Intelligence Scale-Revised (WAIS-R) Block Design Test (BDT) to determine whether visuospatial ability can help discriminate between MCI patients from Dem patients and normal controls (NC). Results showed that the BDT could contribute to the discrimination between MCI and Dem. Specifically, simple BDT task scores could best distinguish MCI from Dem patients, while difficult BDT task scores could contribute to discriminating between MCI and NC. Given the potential clinical value of the BDT in the diagnosis of Dem and MCI, normative data stratified by age and education for the Chinese elderly population are presented for use in research and clinical settings.

## Introduction

Dementia, one of the most common geriatric diseases, greatly affects the quality of life of older adults, bringing with it a series of related economic and public health issues (Weinberger et al., 1993; Zencir et al., 2005). Early detection of dementia and intervention to treat dementia are essential. Researchers have identified a pre-dementia syndrome, named “mild cognitive impairment” (MCI; Petersen et al., 1999). MCI is an intermediate condition lying between normal aging and progression towards Alzheimer’s disease (AD), the most common type of dementia. Patients with MCI will progress to AD at a rate of 10–15% per year, while healthy control subjects who convert at a rate of only 1–2% per year (Petersen et al., 2001; Petersen, 2004).

Research has shown that patients who developed dementia experienced accelerated rates of cognitive decline before diagnosis, and considerable attention has been paid to the area of memory (Rubin et al., 1998; Grober et al., 2000), as an example of cognitive decline. Visuospatial deficits among individuals with MCI and/or Dem have been studied to a much lesser extent than memory has (Iachini et al., 2009). Although some studies found that scores on visuospatial tests did not correlate with the severity of dementia (Kurylo et al., 1996), more studies demonstrated degenerative visuospatial deficits during the progression of dementia (Herlitz et al., 1995; Kaskie and Storandt, 1995; O’Brien et al., 2001; Alegret et al., 2009). The rate of visuospatial ability decline has been found to accelerate in the later stages of dementia (Herlitz et al., 1995), and the stages of visuospatial deficits follow the typical order of memory impairments as dementia progresses. The earliest manifestation of dementia was identified as episodic memory deficit, especially episodic memory disorders (Fox et al., 1998; Wolk and Dickerson, 2011; Romero and Moscovitch, 2012). With the progression of dementia, episodic memory has been found to show a slow decline; while other cognitive functions, such as visuospatial ability, began to show an accelerated decline. Based on the association between visuospatial ability and dementia progression, we expected that visuospatial ability would be able to be used to differentiate between patients with MCI and Dem.

Visuospatial performance in patients with MCI and Dem might change with varying visuospatial task difficulties. Kaskie and Storandt (1995) adopted a simple visuospatial discrimination test to compare Dem patients with healthy controls and found visuospatial deficits in Dem patients. Marcos et al. (2006) found that as impairment toward Dem symptoms progressed, patients with MCI exhibited increased difficulty during complex or demanding visuospatial tasks. In the current study, we will investigate the visuospatial characteristics of MCI and Dem against the backdrop of increasing task difficulty. The Wechsler Adult Intelligence Scale-Revised (WAIS-R) Block Design Test (BDT; Wechsler, 1981) has adaptive difficulty, and is regarded as reflecting of visuospatial ability (Kaufman, 2001). The primary objective of the current study was to explore whether visuospatial ability could make a further contribution, aside from that of episodic memory, in distinguishing patients with MCI patients from those with Dem and from normal controls (NC). We hypothesized that simple visuospatial tasks could help differentiate patients with MCI from those with Dem, and difficult visuospatial tasks could help in the differentiation of patients with MCI from NC.

Given the potential clinical value of measuring visuospatial abilities, the secondary objective of the present study was to collect normative data from a large sample of elderly Chinese individuals, using the BDT (Gong, 1992). The BDT has been found to be effective in diagnosing age-related decline, with performance declining as age increases (Wechsler, 1981; Kaufman et al., 1989), particularly in those aged over 60 years (Rönnlund and Nilsson, 2006). Level of education has also been found to affect BDT performance (Bolton et al., 1966; Ryan et al., 1996; Brooks et al., 2011). Due to the significant potential effects of age and education on BDT performance, and the absence of recent normative data for the BDT, we provide age-and education-adjusted normative data using a large sample of healthy Chinese older adults.

In summary, the current study aims were to (1) determine whether visuospatial ability could help discriminate patients with MCI from those with Dem and from NC; (2) collect normative data from a Chinese elderly population for use in future research and clinical settings.

## Method

### WAIS-R block design test (BDT)

The BDT (Wechsler, 1981) requires that a set of either four or nine, two-colored blocks be arranged so as to duplicate a maximum of 10 target patterns presented in order of ascending difficulty.

This test comprises the following three levels: (1) level 1 (two items), in which participants are asked to arrange a set of four blocks to match that of an experimenter’s illustration, within 60 s of the illustration being created (score range: 0–8); (2) level 2 (four items), in which participants are asked to arrange a set of four blocks according to a presented target pattern, also within 60 s (score range: 0–16); and (3) level 3 (four items), in which participants are asked to arrange a set of nine blocks to duplicate a presented target pattern, within 120 s (score range: 0–24). The tasks in level 1 are relatively the most simple, those in level 2 are of moderate difficulty, and those in level 3 are the most difficult. The maximum aggregated score is 48, with higher scores reflecting better functioning.

### Data collection

This study was conducted in three communities from Chaoyang, Xicheng, and Changping Districts in Beijing. Residents aged 60 and above, who appeared on the census list of the three communities, were contacted for participation. Selection was based on the following inclusion criteria: (1) being aged 60 years old or over, and being registered as permanent residents in their respective residing districts in Beijing (*n* = 1007), and (2) having completed the three measures described below (*n* = 959). The study then excluded individuals (1) whose clinical diagnoses were missing (*n* = 25), and (2) who had received a clinical diagnosis of depression (*n* = 10) according the depression and anxiety subscales of the Structured Clinical Interview for DSM disorders (SCID). The final sample size was 924.

### Procedures and participants

All participants were first informed of the aims and procedures, and assured that their information would remain anonymous and confidential. Each participant signed a voluntary consent form that was approved by the Ethics Committee of the Institute of Psychology at the Chinese Academic of Sciences. Individual demographic information was obtained for each participant before the examination.

Each participant was invited to complete a series of examinations, including a battery of neuropsychological tests, and a clinical assessment. The clinical assessment included items to assess participants’ medical history, a basic physical examination, as well as the Neuropsychiatric Inventory (NPI; Cummings et al., 1994), the Activities of Daily Life (ADL; Katz et al., 1963), the Global Deterioration Scale (GDS; Reisberg et al., 1982), the Clinical Dementia Rating (CDR; Morris, 1993), the Hachinski Ischemic Score (HIS; Hachinski et al., 1975), and the SCID (Spitzer et al., 1985). The neuropsychological battery was administered by undergraduate or graduate research assistants who majored in psychology. The Auditory Verbal Learning Test (Ryan and Geisser, 1986) was used to measure episodic memory ability, with the number of words recalled after a delay interval of 30 min was taken as an indicator of delayed recall (DR). The Mini Mental State Examination (MMSE) was administered to assess global cognition among the participants; this test was scored according to the procedures described in the original paper (Folstein et al., 1975).

All research assistants and clinicians were intensively trained. High inter-rater reliability (above 90%) was obtained with the support of a consensus diagnosis meeting at which the neuropsychological and clinical data were reviewed. The screening process was standardized with a comprehensive Case Report Form, on which each participant’s results were recorded. Experienced neurologists performed all clinical diagnoses. Eighteen patients were diagnosed with dementia, based on the Diagnostic and Statistical Manual of Mental Disorders, Fourth Edition (DSM-IV, 1996), including 12 with AD and six with vascular dementia. Participants that met the following criteria of Peterson (2004) were diagnosed with MCI: (1) preserved general cognitive function as confirmed by meeting an education-based criterion of the MMSE, specifically, an MMSE score of ≥24 for those who had received more than or equal to 7 years of education, ≥20 for those who had received less than 7 years of education, and ≥17 for those who were illiterate; (2) being ≥1.5 SD below the sample mean scores on tests in at least one cognitive domain within the areas of episodic memory, language, executive function, or visuospatial skills; (3) a global CDR score of 0.5; (4) receiving a result of level 2 or level 3 in the GDS; (5) intact ADL ratings; and (6) an absence of dementia. Sixty individuals were diagnosed with MCI, including 48 with amnestic MCI and 12 with non-amnestic MCI. The other 846 individuals were considered as NC.

The 846 participants in the NC group were aged 60–93 years (*M* = 70.12, SD = 6.99) and had education levels ranging from 0 to 24 years (*M* = 10.65, SD = 5.23). The mean MMSE score for this group was 26.80 (SD = 3.53). The gender ratio (males/females) was approximately 45/55 (380/466).

### Statistical analysis

Group differences in demographic variables, and neuropsychological test results were examined by using one-way analysis of variance (ANOVA) or a chi-square analysis. Moreover, as statistically significant differences in age and education were found among the three diagnosed groups, non-parametric analysis of covariance (rank ANCOVA) was used to compare scores on the BDT among the diagnosed groups (NC/MCI/Dem), adjusting for age and level of education. *Post hoc* analyses were further performed with the significant level adjusted by the Bonferroni method.

Binary logistic regression was conducted respectively to evaluate the contribution of the BDT in differentiating individuals with MCI from those with dementia, and those with MCI from NC. In model 1, we entered age, level of education, and the DR score into the regression equation. In model 2, we added the total score of the BDT plus the variables in model 1 into the equation. In models 3, 4, and 5, we added the scores of BDT levels 1, 2, and 3, respectively, plus the variables in model 1 into the equation. To compare models 1 and 2, we were able to investigate the additional contribution of the BDT, other than DR in differentiating patients with MCI from those with Dem and NC. Through the comparison of models 3 to 5, we were able to find which level of the BDT was most effective in differentiating patients with MCI from those with Dem, and patients with MCI from NC. Receiver operating characteristic (ROC) analysis was used to assess the effectiveness of each level of BDT in differentiating MCI from NC and Dem.

The second aim of the current study was to provide normative data for the Chinese elderly population. Due to the potential significant contributions of demographic variables to BDT performance, stepwise multiple linear regression analyses were employed among the NC group to demonstrate probable correlations between the demographic variables (age, level of education, and gender) and measures of BDT. Given that BDT performance correlates with age and level of education, participants were divided into five age groups (60–64, 65–69, 70–74, 75–79, and 80–95), each with three educational levels (≤8, 9–12, and ≥13 years of education).

All statistical analyses were conducted using SPSS version 19.0 (IBM Corporation, Somers, NY).

## Results

### Demographic characteristics and group differences

The sample’s demographic characteristics are summarized in Table 1. There were significant differences among NC, MCI and AD with respect to age (*F*_(2,920)_ = 9.22, *p* < 0.001) and level of education (*F*_(2,919)_ = 25.76, *p* < 0.001), with patients with AD being older than those in the MCI and NC groups, and NC participants more highly educated than those in the AD and MCI groups. The proportion of men and women did not differ among groups (*χ*^2^ = 4.48, *df* = 2, *p* = 0.110).

**Table 1:** **Demographic characteristics and mean scores on the neuropsychological tests (*M* ± SD)**.

	NC (*N* = 846)	MCI (*N* = 60)	Dem (*N* = 18)	*p*-value
age	70.12 ± 6.99	71.40 ± 7.29	77.00 ± 7.08	<0.001^a^
%female	55.0%	58.3%	77.8%	0.20
years of education	10.66 ± 5.22	7.75 ± 4.53	3.33 ± 3.97	<0.001^b^
ADL	14.40 ± 2.50	15.12 ± 4.10	27.38 ± 10.15	<0.001^a^
MMSE	26.83 ± 3.47	24.45 ± 3.68	12.83 ± 5.50	<0.001^b^
DR	9.04 ± 4.32	5.57 ± 3.99	1.11 ± 2.11	<0.001^b^
BDT	26.94 ± 9.53	21.05 ± 8.38	6.78 ± 6.80	<0.001^b^
Adjusted mean ± SE^d^	26.57 ± 0.26	23.82 ± 0.99	15.04 ± 1.84
BDT Level 1	6.87 ± 1.86	6.30 ± 2.01	1.89 ± 2.32	<0.001^a^
Adjusted mean ± SE^d^	6.83 ± 0.06	6.65 ± 0.23	2.91 ± 0.42
BDT Level 2	13.80 ± 3.64	11.87 ± 4.03	4.89 ± 5.75	<0.001^b^
Adjusted mean ± SE^d^	13.69 ± 0.11	12.80 ± 0.42	7.69 ± 0.77
BDT Level 3	6.29 ± 6.10	2.88 ± 4.54	0	<0.001^c^
Adjusted mean ± SE^d^	6.09 ± 0.18	4.40 ± 0.67	4.48 ± 1.24

*Notes: NC = Normal Controls; MCI = Mild Cognitive Impairment; Dem = Dementia; MMSE = Mini-mental State Examination; ADL = Ability of Daily Living; BDT = Block design test; DR = Delayed Recall*.

*^a^Dem < NC = MCI; ^b^Dem < MCI < NC ; ^c^Dem = MCI < NC; ^d^p-value obtained from non-parametric analysis of covariance with adjustment for age and education*.

Group differences were found for the total score (*F*_(2,921)_ = 49.21, *p* < 0.001) and each level of the BDT (*ps* < 0.01). Results remained significant when further analyzed with age and level of education as covariates. *Post hoc* analyses showed that for the BDT (*F*_(2,916)_ = 21.72, *p* < 0.001), the performance of the MCI group was better than that of the Dem group (*t* = 4.24, *p* < 0.001) and worse than that of the NC group (*t* = 2.68, *p* = 0.007). Specifically, for level 1 (*F*_(2,914)_ = 42.70, *p* < 0.001), the performance of the MCI group was similar to that of the NC group (*t* = 0.84, *p* = 0.404), and significantly better than that of the Dem group (*t* = 7.93, *p* < 0.001); for level 2 (*F*_(2,914)_ = 30.88, *p* < 0.001), the performance of the MCI group was worse than that of the NC group (*t* = 2.06, *p* = 0.040), and significantly better than that of the Dem group (*t* = 5.90, *p* < 0.001); while for level 3 (*F*_(2,914)_ = 3.59, *p* = 0.028), the performance of the MCI group was significantly worse than that of the NC group (*t* = 2.44, *p* = 0.015), and similar to that of the Dem group (*t* = 0.06, *p* = 0.954).

### The effectiveness of the BDT in differentiating individuals with MCI from those with dementia and NC

Binary logistic regression analysis was conducted to assess the contribution of the BDT in distinguishing patients with MCI from those with Dem (Table 2). In model 2, the BDT score was indicated as a significant predictor (*p* = 0.003) in the differentiation between MCI and Dem groups. Compare models 1 and 2, we found that the BDT would make an additional contribution (Δ*R*^2^ = 0.168, *p* < 0.001) other than DR in the discrimination between MCI and Dem groups. When we compared models 3 to 5, we found that the BDT level 1 (*p* = 0.002) and BDT level 2 (*p* = 0.011) scores were significant predictors, while the BDT level 3 score was not a significant predictor (*p* = 0.997). These results showed that the BDT could contribute to the discrimination between MCI and Dem groups, and that the score on simple BDT tasks (BDT levels 1 and 2) could significantly distinguish patients with MCI from those with Dem.

**Table 2:** **Summary of logistic regression analysis in differentiating MCI from NC and Dem**.

		MCI vs. NC					MCI vs. Dem				
Model	Variables^a^	B^b^	S.E.	Exp (95%CI)^c^	*p*	*R*^2^	B^b^	S.E.	Exp (95%CI)^c^	*p*	*R*^2^
1	Age	−0.019	0.020	0.981 (0.944–1.019)	0.326	0.106	0.019	0.046	1.020 (0.931–1.117)	0.674	0.463
	Education	−0.062	0.028	0.940 (0.889–0.994)	0.030		−0.190	0.089	0.827 (0.695–0.983)	0.032
	DR	−0.150	0.033	0.860 (0.806–0.918)	0.000		−0.385	0.141	0.681 (0.516–0.898)	0.006
2	Age	−0.022	0.020	0.978 (0.941–1.016)	0.255	0.114	0.016	0.059	1.016 (0.905–1.141)	0.790	0.631
	Education	−0.034	0.033	0.967 (0.906–1.031)	0.304		−0.115	0.113	0.892 (0.715–1.112)	0.308
	DR	−0.141	0.034	0.869 (0.812–0.929)	0.000		−0.248	0.169	0.781 (0.561–1.087)	0.142
	BDT	−0.029	0.018	0.971 (0.939–1.005)	0.098		−0.189	0.064	0.827 (0.730–0.938)	0.003
3	Age	−0.019	0.020	0.981 (0.944–1.019)	0.325	0.106	0.025	0.066	1.026 (0.902–1.166)	0.699	0.656
	Education	−0.063	0.030	0.939 (0.886–0.996)	0.035		−0.081	0.110	0.923 (0.744–1.144)	0.463	
	DR	−0.151	0.034	0.860 (0.805–0.918)	0.000		−0.287	0.172	0.751 (0.536–1.052)	0.096
	BDT level 1	0.006	0.067	1.006 (0.882–1.148)	0.924		−0.593	0.190	0.553 (0.381–0.082)	0.002
4	Age	−0.021	0.020	0.979 (0.942–1.018)	0.287	0.108	0.019	0.054	1.019 (0.916–1.132)	0.732	0.559
	Education	−0.051	0.032	0.950 (0.893–1.011)	0.108		−0.171	0.103	0.843 (0.689–1.031)	0.096
	DR	−0.147	0.034	0.863 (0.808–0.922)	0.000		−0.285	0.153	0.752 (0.557–1.014)	0.062
	BDT level 2	−0.028	0.035	0.972 (0.908–1.041)	0.421		−0.187	0.074	0.829 (0.717–0.958)	0.011
5	Age	−0.023	0.020	0.977 (0.940–1.015)	0.233	0.124	−0.028	0.051	0.972 (0.879–1.075)	0.585	0.555
	Education	−0.026	0.032	0.974 (0.915–1.037)	0.411		−0.128	0.103	0.880 (0.720–1.076)	0.212
	DR	−0.143	0.034	0.866 (0.811–0.926)	0.000		−0.457	0.166	0.633 (0.457–0.877)	0.006
	BDT level 3	−0.087	0.038	0.917 (0.851–0.988)	0.022		−4.664	1077.60	0.009 (0–)	0.997	

*Note: ^a^variables entered into models 1 to 5; ^b^original coefficient for each variable obtained from the logistic regression analysis; ^c^exponentiated coefficient and its 95% confidence interval for each variable obtained from the logistic regression analysis. Exponentiated coefficient less than 1 reflects negative relationships while value above 1 denotes positive relationship*.

Binary logistic regression analysis was used to assess the effectiveness of the BDT in differentiating patients with MCI from NC (Table 2). In model 2, the score on the BDT could not significantly differentiate patients with MCI from NC (*p* = 0.098). When compared models 1 and 2, we found that the BDT could not make an additional contribution (Δ*R*^2^ = 0.008, *p* = 0.097) other than DR in the discrimination between MCI and NC groups. Compare models 3 to 5, we found that only the score on BDT level 3 was a significant predictor (*p* = 0.022) in differentiating patients with MCI from NC. These results showed that the difficult BDT tasks (BDT level 3) could contribute to discriminating between patients with MCI and NC.

**Figure 1:**
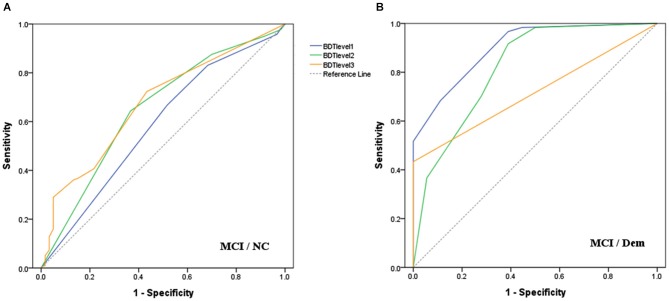
**Receiver operating characteristic curves for each level of BDT to detect: **(A)** MCI from NC; **(B)** MCI from Dem**.

ROC curves (Figure 1) were drawn to determine the discriminatory validity of each level of BDT for MCI vs. Dem groups, as well as MCI vs. NC groups. The area under the curve (AUC) of BDT level 3 (0.67, 95% CI: 0.61–0.74) was the largest for the discrimination between MCI and NC groups (Figure 1A). With regard to the discrimination between MCI and Dem groups, BDT level 1 (0.91, 95% CI: 0.83–0.98) demonstrated the largest AUC in comparison to the other levels of BDT (Figure 1B).

### Normative data of the BDT in NC

Stepwise multiple linear regression analyses revealed that, age and level of education significantly predicted BDT scores (*ps* < 0.010). However, there was no significant effect of gender on BDT performance (*ps* > 0.050). Table 3 depicts normative data for each of the 15 age groups according to level of education.

**Table 3:** **Normative data on the BDT stratified by age and education among NC (*M*, SD)**.

			BDT		Level 1		Level 2		Level 3	
Age	Education	*N*	*M*	SD	*M*	SD	*M*	SD	*M*	SD
60–64	0–8	40	20.75	8.14	5.85	2.58	11.90	3.89	3.00	3.19
	9–12	122	28.00	8.73	7.07	1.65	14.33	2.90	6.57	6.17
	13–	72	33.69	7.25	7.67	1.01	15.50	1.49	10.53	6.40
	Total	234	28.51	9.25	7.04	1.79	14.27	3.00	7.18	6.37
65–69	0–8	35	22.40	9.81	6.11	2.65	12.69	4.39	3.60	5.17
	9–12	73	26.71	8.01	7.04	1.67	14.08	3.06	5.59	5.28
	13–	69	34.04	6.77	7.68	0.81	15.65	1.14	10.71	6.34
	Total	177	28.72	9.12	7.11	1.76	14.42	3.05	7.19	6.37
70–74	0–8	35	20.89	8.77	5.89	2.05	11.77	5.22	3.23	4.04
	9–12	53	25.04	6.91	6.75	1.63	13.96	2.67	4.32	4.62
	13–	120	32.67	7.15	7.47	1.09	15.43	1.49	9.78	6.14
	Total	208	28.75	8.77	7.02	1.55	14.44	3.06	7.28	6.19
75–79	0–8	63	19.60	8.18	6.41	2.13	11.11	4.95	2.08	3.17
	9–12	36	23.86	7.70	6.72	1.67	13.11	3.53	4.03	4.78
	13–	43	29.53	7.70	7.40	1.28	15.16	1.65	7.63	5.36
	Total	142	23.69	8.93	6.79	1.83	12.89	4.20	4.25	4.92
80–	0–8	54	15.54	8.45	5.22	2.65	9.19	5.13	1.13	2.15
	9–12	18	25.00	6.47	6.56	1.65	14.00	2.47	4.44	4.44
	13–	13	28.85	7.83	6.62	2.36	15.08	1.75	7.15	4.96
	Total	85	19.58	9.61	5.72	2.49	11.11	4.99	2.29	3.96
Total	0–8	227	19.47	8.84	5.90	2.43	11.14	4.89	2.43	3.59
	9–12	302	26.50	8.11	6.93	1.69	14.04	2.97	5.51	5.52
	13–	317	32.62	7.33	7.51	1.14	15.44	1.47	9.75	6.17
	Total	846	26.91	9.57	6.87	1.86	13.79	3.64	6.27	6.10

## Discussion

The results confirmed our hypothesis that the BDT could help discriminate between individuals with MCI and those with Dem. In particular, the score on simple BDT tasks was the best for distinguishing patients with MCI from those with Dem, and the score on difficult BDT tasks could contribute to discriminating between patients with MCI and NC.

Visuospatial ability was effective in discriminating patients with MCI from those with Dem. Episodic memory seems to show an accelerated decline during the early stages of MCI (Hall et al., 2001); however, with the progression of dementia, visuospatial ability shows larger declines than does memory. It is assumed that memory impairment might have reached its limit at a certain stage of the disease. Yu et al. (2012) drew ROC curves to determine the discriminatory validity of seven cognitive domains of the MoCA for patients with MCI vs. NC, and for patients with MCI vs. Dem. The study found that the most sensitive domains with regard to the discrimination between patients with MCI and Dem were the orientation and visuospatial/executive domains (see Nasreddine et al., 2005). These findings indicated that visuospatial abilities might be a more sensitive predictor in the discrimination between patients with MCI and those with Dem, also making a larger contribution towards such discrimination. Furthermore, scores on the BDT level 1 were significant in differentiating patients with MCI from those with Dem, with a high AUC found (0.91, 95% CI: 0.83–0.98), and this measure can be completed in a very short time. This suggests that the BDT level 1 measure could be applied as an effective tool in the diagnosis of Dem for clinical use, with the advantages of high diagnostic accuracy and time efficiency. In addition, visuospatial performance of patients with MCI and those with Dem changed with varying visuospatial task difficulties. Future studies must consider the difficulty and type of tasks when assessing visuospatial ability in patients with cognitive impairment, because increasing the difficulty of a visuospatial task could lead to an increase in the diagnosis of more subtle cognitive deficits (i.e., MCI).

Given the potential clinical value of visuospatial abilities in the diagnosis of Dem and MCI, normative data stratified by age and level of education were collected for use in future research and clinical settings. In the present study, BDT performance declined with age, which is consistent with previous research findings (Rönnlund and Nilsson, 2006). This age-related decline in BDT performance indicated that visuospatial ability degraded with normal aging. Level of education was positively correlated with BDT performance, such that less educated participants performed worse on the BDT. Higher education might be related to participation in more intellectual activities during leisure time, which might in turn protect aging individuals against dementia (Kliegel et al., 2004). Furthermore, the normative information presented in this study could serve as assessment criteria. As such, this study contributes to the development of appropriate neuropsychological test norms for the Chinese population. As age and level of education were significantly correlated with BDT performance, these two demographic factors should be taken into consideration when interpreting BDT scores.

The current study highlighted the contribution of BDT in discriminating MCI from Dem and provided a potential diagnostic tool of Dem for research and clinical use in the field of age-related cognitive impairment. The present findings also constitute a significant contribution to the expanding knowledge on age-related changes in visuospatial ability by providing normative data sourced from a large sample of older adults. These findings have potential future clinical utility in that they provide clinicians with information on normative differences across education levels and age. Nevertheless, there are several limitations that should be acknowledged. First, this was a cross-sectional study; therefore, age differences in performance on the BDT might have been exacerbated by factors that are associated with the use of cohorts. Furthermore, we could not directly analyze the predictive accuracy of the BDT score on the conversion rate of MCI to dementia. Future research could assess the value of the BDT score in the differential diagnosis, prognosis, and conversion prediction of MCI to dementia by recruiting participants from more diverse regions or countries. Second, we did not distinguish between different types of dementia. Patients with different types of dementia may demonstrate varying patterns of visuospatial performance (Heyanka et al., 2010); consequently, the ability of visuospatial ability to discriminate between MCI and dementia may vary across different types of dementia. Third, the sample sizes of the NC, MCI, and dementia groups were different. The number of dementia patients was quite small, which might have compromised the statistical potency of between-group comparisons. Lastly, the current study presented cross-sectional data with respect to BDT performance, but longitudinal normative data should be developed to investigate the cognitive trajectory of normal aging and neurodegenerative diseases, and determine the clinical and experimental significance of longitudinal changes on this measure.

## Conflict of interest statement

The authors declare that the research was conducted in the absence of any commercial or financial relationships that could be construed as a potential conflict of interest.

## References

[B1] Alegret M. Boada-Rovira M. Vinyes-Junqué G. Valero S. Espinosa A. Hernández I.. (2009). Detection of visuoperceptual deficits in preclinical and mild Alzheimer’s disease. J. Clin. Exp. Neuropsychol. 31, 860–867. 10.1080/13803390802595568 19142775 PMC2834652

[B36] Bolton N. Britton P. Savage R. (1966). Some normative data on the WAIS and its indices in an aged population. J. Clin. Psychol. 22, 184–188. 10.1002/1097-4679(196604)22:2<184::aid-jclp2270220217>3.0.co;2-n 5937042

[B38] Brooks B. L. Holdnack J. A. Iverson G. L. (2011). Advanced clinical interpretation of the WAIS-IV and WMS-IV: prevalence of low scores varies by level of intelligence and years of education. Assessment 18, 156–167. 10.1177/1073191110385316 20947705

[B2] Cummings J. L. Mega M. Gray K. Roseberg-Thompson S. Carusi D. A. Gornbein J. (1994). The neuropsychiatric inventory: comprehensive assessment of psychopathology in dementia. Neurology 44, 2308–2314. 10.1212/WNL.44.12.2308 7991117

[B3] Folstein M. F. Folstein S. E. McHugh P. R. (1975). Mini-mental state: a practical method for grading the cognitive state of patients for the clinician. J. Psychiatr. Res. 12, 189–198. 10.1016/0022-3956(75)90026-6 1202204

[B4] Fox N. Warrington E. Seiffer A. Agnew S. Rossor M. (1998). Presymptomatic cognitive deficits in individuals at risk of familial Alzheimer’s disease. A longitudinal prospective study. Brain 121, 1631–1639. 10.1093/brain/121.9.1631 9762953

[B5] Gong Y. (1992). Manual of Wechsler Adult Intelligence Scale-Chinese Version. Changsha: Chinese Map Press.

[B6] Grober E. Lipton R. B. Hall C. Crystal H. (2000). Memory impairment on free and cued selective reminding predicts dementia. Neurology 54, 827–832. 10.1212/wnl.54.4.827 10690971

[B7] Hachinski V. C. Iliff L. D. Zilhka E. Du Boulay G. H. McAllister V. L. Marshall J.. (1975). Cerebral blood flow in dementia. Arch. Neurol. 32, 632–637. 10.1001/archneur.1975.00490510088009 1164215

[B8] Hall C. B. Ying J. Kuo L. Sliwinski M. Buschke H. Katz M.. (2001). Estimation of bivariate measurements having different change points, with application to cognitive ageing. Stat. Med. 20, 3695–3714. 10.1002/sim.1113 11782027

[B9] Herlitz A. Hill R. D. Fratiglioni L. Bäckman L. (1995). Episodic memory and visuospatial ability in detecting and staging dementia in a community-based sample of very old adults. J. Gerontol. A Biol. Sci. Med. Sci. 50, M107–M113. 10.1093/gerona/50a.2.m107 7874587

[B10] Heyanka D. J. Mackelprang J. L. Golden C. J. Marke C. D. (2010). Distinguishing Alzheimer’s disease from vascular dementia: an exploration of five cognitive domains. Int. J. Neurosci. 120, 409–414. 10.3109/00207451003597177 20504211

[B11] Iachini I. Iavarone A. Senese V. P. Ruotolo F. Ruggiero G. (2009). Visuospatial memory in healthy elderly, AD and MCI: a review. Curr. Aging Sci. 2, 43–59. 10.2174/1874612810902010043 20021398

[B12] Kaskie B. Storandt M. (1995). Visuospatial deficit in dementia of the Alzheimer type. Arch. Neurol. 52, 422–425. 10.1001/archneur.1995.00540280120025 7710379

[B28] Katz S. Ford A. B. Moskowitz R. W. Jackson B. A. Jaffe M. W. (1963). Studies of illness in aged: the index of ADL: a standard measure of biological and psychological function. JAMA 185, 914–919. 10.1001/jama.1963.03060120024016 14044222

[B13] Kaufman A. S. (2001). WAIS-III IQs, Horn’s theory and generational changes from young adulthood to old age. Intelligence 29, 131–167 10.1016/s0160-2896(00)00046-5

[B35] Kaufman A. S. Reynolds C. R. McLean J. E. (1989). Age and WAIS-R intelligence in a national sample of adults in the 20-to 74-year age range: a cross-sectional analysis with educational level controlled. Intelligence 13, 235–253 10.1016/0160-2896(89)90020-2

[B14] Kliegel M. Zimprich D. Rott C. (2004). Life-long intellectual activities mediate the predictive effect of early education on cognitive impairment in centenarians: a retrospective study. Aging Ment. Health 8, 430–437. 10.1080/13607860410001725072 15511741

[B15] Kurylo D. D. Corkin S. Rizzo J. F. Growdon J. H. (1996). Greater relative impairment of object recognition than of visuospatial abilities in Alzheimer’s disease. Neuropsychology 10, 74–81 10.1037/0894-4105.10.1.74

[B16] Marcos A. Gil P. Barabash A. Rodriguez R. Encinas M. Fernández C.. (2006). Neuropsychological markers of progression from mild cognitive impairment to Alzheimer’s disease. Am. J. Alzheimers Dis. Other Demen. 21, 189–196. 10.1177/1533317506289348 16869340 PMC10833278

[B17] Morris J. C. (1993). The Clinical Dementia Rating (CDR): current version and scoring rules. Neurology 43, 2412–2414. 10.1212/wnl.43.11.2412-a 8232972

[B18] Nasreddine Z. S. Phillips N. A. Bédirian V. Charbonneau S. Whitehead V. Collin I.. (2005). The Montreal Cognitive Assessment, MoCA: a brief screening tool for mild cognitive impairment. J. Am. Geriatr. Soc. 53, 695–699. 10.1111/j.1532-5415.2005.53221.x 15817019

[B19] O’Brien H. L. Tetewsky S. J. Avery L. M. Cushman L. A. Makous W. Duffy C. J. (2001). Visual mechanisms of spatial disorientation in Alzheimer’s disease. Cereb. Cortex 11, 1083–1092. 10.1093/cercor/11.11.1083 11590117

[B20] Petersen R. C. (2004). Mild cognitive impairment as a diagnostic entity. J. Intern. Med. 256, 183–194. 10.1111/j.1365-2796.2004.01388.x 15324362

[B21] Petersen R. C. Doody R. Kurz A. Mohs R. C. Morris J. C. Rabins P. V.. (2001). Current concepts in mild cognitive impairment. Arch. Neurol. 58, 1985–1992. 10.1001/archneur.58.12.1985 11735772

[B22] Petersen R. C. Smith G. E. Waring S. C. Ivnik R. J. Tangalos E. G. Kokmen E. (1999). Mild cognitive impairment: clinical characterization and outcome. Arch. Neurol. 56, 303–308. 10.1001/archneur.56.3.303 10190820

[B23] Reisberg B. Ferris S. H. de Leon M. J. Crook T. (1982). The global deterioration scale for assessment of primary degenerative dementia. Am. J. Psychiatry 139, 1136–1139. 10.1176/ajp.139.9.1136 7114305

[B24] Romero K. Moscovitch M. (2012). Episodic memory and event construction in aging and amnesia. J. Mem. Lang. 67, 270–284 10.1016/j.jml.2012.05.002

[B25] Rönnlund M. Nilsson L. G. (2006). Adult life-span patterns in WAIS-R block design performance: cross-sectional versus longitudinal age gradients and relations to demographic factors. Intelligence 34, 63–78 10.1016/j.intell.2005.06.004

[B26] Rubin E. H. Storandt M. Miller J. P. Kinscherf D. A. Grant E. A. Morris J. C.. (1998). A prospective study of cognitive function and onset of dementia in cognitively healthy elders. Arch. Neurol. 55, 395–401. 10.1001/archneur.55.3.395 9520014

[B37] Ryan J. J. Dai X. Lopez S. J. (1996). Intersubtest scatter on the Wechsler Adult Intelligence Scale—Revised for China: Reply to Li and Balfour (1996). Psychol. Assess. 8, 102–104 10.1037/1040-3590.8.1.102

[B27] Ryan J. J. Geisser M. E. (1986). Validity and diagnostic accuracy of an alternate form of the Rey auditory verbal learning test. Arch. Clin. Neuropsychol. 1, 209–217. 10.1093/arclin/1.3.209 14591149

[B29] Spitzer R. L. Gibbon M. Williams J. B. (1985). Instruction Manual for Structured Clinical Interview for DSM-III-R (SCID). New York: State Psychiatric Institute.

[B30] Wechsler D. (1981). WAIS-R manual: Wechsler Adult Intelligence Scale-Revised. New York: Jovanovich HB. Psychological Corporation.

[B31] Weinberger M. Gold D. T. Divine G. W. Cowper P. A. Hodgson L. G. Schreiner P. J.. (1993). Expenditures in caring for patients with dementia who live at home. Am. J. Public Health 83, 338–341. 10.2105/ajph.83.3.338 8438969 PMC1694663

[B32] Wolk D. A. Dickerson B. C. Alzheimer’s Disease Neuroimaging Initiative. (2011). Fractionating verbal episodic memory in Alzheimer’s disease. Neuroimage 54, 1530–1539. 10.1016/j.neuroimage.2010.09.005 20832485 PMC2997155

[B33] Yu J. Li J. Huang X. (2012). The Beijing version of the montreal cognitive assessment as a brief screening tool for mild cognitive impairment: a community-based study. BMC Psychiatry 12:156. 10.1186/1471-244X-12-156 23009126 PMC3499377

[B34] Zencir M. Kuzu N. Beşer N. G. Ergin A. Çatak B. Şahiner T. (2005). Cost of Alzheimer’s disease in a developing country setting. Int. J. Geriatr. Psychiatry 20, 616–622. 10.1002/gps.1332 16021668

